# Molecular Characterization and In Vitro Functional Analysis of a 1-Cys Peroxiredoxin 6 from the Whiteleg Shrimp *Penaeus vannamei*

**DOI:** 10.3390/genes17040428

**Published:** 2026-04-06

**Authors:** Gunasekara Chathura Wikumpriya, W. S. P. Madhuranga, Chan-Hee Kim

**Affiliations:** Major in Aquaculture and Applied Life Sciences, Division of Fisheries Life Science, College of Fisheries Science, Pukyong National University, Busan 48513, Republic of Korea

**Keywords:** *Penaeus vannamei*, 1-Cys peroxiredoxin 6, antioxidant defense, innate immunity

## Abstract

Background/Objectives: Peroxiredoxins (Prxs) are key antioxidant enzymes involved in cellular redox homeostasis. Prx6 is a multifunctional member of the Prx family that has been reported in other organisms to possess glutathione peroxidase and phospholipase A_2_ (PLA_2_)-related activities. However, the structural and immunological roles of 1-Cys Prx6 in crustaceans remain poorly understood. This study aimed to identify and characterize a Prx6 gene from *Penaeus vannamei* (*Pv*Prx6) and to evaluate its potential involvement in antioxidant defense. Methods: *Pv*Prx6 cDNA was identified and analyzed using bioinformatics and AlphaFold2 modeling. Tissue distribution and transcriptional responses to lipopolysaccharide (LPS), poly(I:C), and peptidoglycan (PGN) were examined by RT-qPCR. Recombinant *Pv*Prx6 (r*Pv*Prx6) was expressed in *Escherichia coli*, and its antioxidant activity was evaluated in vitro using a metal-catalyzed oxidation (MCO) assay. Results: *Pv*Prx6 encodes a 219-amino-acid protein containing conserved AhpC/TSA and 1-Cys Prx domains. Sequence comparison and 3D modeling revealed conserved peroxidase (Thr^41^, Cys^44^, Arg^127^) and residues (His^23^, Lys^29^, Asp^135^) corresponding to the reported PLA_2_-associated motif. Structural analysis suggested that Lys^29^ occupies a position corresponding to the Ser^32^ residue of human Prx6, although this did not imply functional equivalence. *Pv*Prx6 transcripts were highly expressed in the lymphoid organ and hepatopancreas and were significantly induced at 12 h following immune challenge. r*Pv*Prx6 exhibited dose-dependent protection against hydroxyl radical-mediated DNA damage under the experimental conditions. Conclusions: Collectively, these findings suggest that *Pv*Prx6 retains conserved structural characteristics of Prx6 proteins and may contribute to antioxidant defense in *P. vannamei*. However, further studies are required to validate its enzymatic activity and in vivo functional roles.

## 1. Introduction

Reactive oxygen species (ROS) are generated as byproducts of normal cellular metabolism, primarily within the mitochondrial electron transport chain during oxidative phosphorylation, and are also produced by immune cells during the oxidative burst to combat pathogens [1,2]. At physiological levels, ROS participate in multiple cellular processes, including cell signaling and host defense [3,4,5]. However, excessive ROS can react with various biological molecules, including proteins, lipids, and nucleic acids, leading to irreversible cell damage and potentially resulting in cell death [6,7,8]. Aerobic organisms therefore possess antioxidant defense systems that tightly regulate intracellular ROS levels, including enzymes such as superoxide dismutase (SOD), catalase (CAT), glutathione peroxidase (GPx), and peroxiredoxins (Prxs), which work synergistically to maintain cellular redox balance [9,10,11,12,13].

Among these, Prxs are a superfamily of non-selenium thiol peroxidases conserved across all kingdoms of life [2,11,14]. In mammals, six Prx isoforms (Prx1–Prx6) have been identified and categorized into two subfamilies based on the number of cysteine residues involved in their catalytic mechanisms: the 2-Cys Prxs (Prx1–5) and the 1-Cys Prx (Prx6) [14,15]. These isoforms exhibit distinct subcellular localizations associated with their specific functions; for instance, Prx1, Prx2, and Prx6 are primarily localized in the cytosol, while others are restricted to the mitochondria (Prx3), endoplasmic reticulum (Prx4), or peroxisomes (Prx5) [15,16]. The 2-Cys Prxs utilize a conserved peroxidatic cysteine (*C_P_*) and a resolving cysteine (*C_R_*) to form a disulfide bond, subsequently reduced by the thioredoxin (Trx) system [15,17]. In contrast, Prx6, the sole 1-Cys Prx member, contains only a single conserved *C_P_* that is oxidized to cysteine sulfenic acid (*C_P_-SOH*) and directly reduced back to its thiol form by glutathione (GSH) and glutathione S-transferase (GST) [18].

Beyond its unique catalytic cycle, Prx6 is a multifunctional enzyme. It is the only Prx capable of binding and reducing phospholipid hydroperoxides, thereby protecting cell membrane integrity [18,19]. Furthermore, it exhibits calcium-independent phospholipase A_2_ (*i*PLA_2_) activity, which is crucial for phospholipid turnover and cellular signaling [18,20]. Prx6 also plays an important role in protecting cells from oxidative damage and maintaining redox homeostasis [18,21].

In aquatic organisms, Prx6-related antioxidant responses have been associated with environmental stress conditions, such as hypothermal challenge in milkfish, and immune responses to bacterial infection in crustaceans [22,23]. Consistent with its role in redox regulation, ROS in aquatic invertebrates, including shrimp, are not only potentially harmful oxidants but also important mediators of innate immune responses [2,23,24]. Shrimp rely exclusively on innate immunity, in which ROS function as antimicrobial effectors and signaling molecules during responses to environmental stress and pathogen challenge [25,26]. To prevent excessive oxidative damage, antioxidant defense systems composed of enzymes such as SOD, CAT, GPx, and Prx family members must be tightly regulated [3,27]. Previous studies have shown that antioxidant-related genes in shrimp are dynamically modulated during bacterial and viral infections, highlighting the importance of redox homeostasis in crustacean immunity [3,26,28].

In the context of aquaculture, the whiteleg shrimp *Penaeus vannamei* is the most economically significant crustacean species, representing a major proportion of global shrimp aquaculture production, with substantial economic value [29]. However, the industry faces severe threats from infectious diseases such as acute hepatopancreatic necrosis disease (AHPND) caused by *Vibrio parahaemolyticus* and white spot syndrome virus (WSSV) [30,31].

Recent studies have identified multiple Prx family members in crustaceans, including shrimp and crabs, where they are associated with antioxidant defense and immune responses under environmental stress and pathogen challenges [23,32,33]. In shrimp, several Prx isoforms have been shown to be differentially expressed in response to bacterial and viral infections, suggesting their potential roles in maintaining redox homeostasis during immune activation. For instance, Prx4 has been shown to enhance prophenoloxidase (proPO) activity and upregulate Toll pathway-related genes following AHPND infection [34]. Similarly, Prx2 (or Prx2-like) is reported to regulate the mRNA expression of key immune effectors, including crustin, anti-lipopolysaccharide factor 3 (ALF3), and CAT, in response to WSSV challenge [35]. These findings suggest that Prx proteins may contribute to shrimp immune responses. To the best of our knowledge, however, the specific physiological and immunological roles of Prx6 in shrimp species remain poorly understood.

In this study, we hypothesized that *Pv*Prx6 contributes to antioxidant defense during immune activation in *P. vannamei*. To address this hypothesis, we performed molecular characterization, expression profiling under immune stimulation, and an in vitro assessment of the protective activity of recombinant *Pv*Prx6. This study provides a species-specific molecular and biochemical characterization of *Pv*Prx6 and offers a basis for future functional investigations of its role in shrimp immunity.

## 2. Materials and Methods

### 2.1. Shrimp Rearing and Tissue Collection

Healthy juvenile whiteleg shrimp (*P. vannamei*, one-month-old) were purchased from a commercial shrimp farm located at Muan-gun, Jeollanam-do, and transported to the laboratory at Pukyong National University in Busan, South Korea. At the time of purchase, shrimp had an average body weight of 0.9 ± 0.1 g and a total length of 1.8 ± 0.2 cm. They were subsequently maintained under laboratory conditions and allowed to acclimate and grow until they reached an average body weight of 3.0 ± 0.5 g and a total length of 2.5 ± 0.3 cm, at which point they were used for experiments. The shrimp were reared in an indoor recirculating tank (1.0 × 3.0 × 0.5 m; width × length × height) using natural seawater (30–32 ppt) filtered through a sponge filter and UV sterilization system, with continuous aeration at 24 ± 1 °C under a 12 h light:12 h dark photoperiod. They were fed four times daily with a commercial diet to apparent satiation (Jeil Feed Co., Ltd., Daejeon, Republic of Korea). Water quality was monitored daily during the rearing period, and the following parameters were maintained: dissolved oxygen (DO) at 9.7 ± 0.2 mg/L; pH at 7.3–7.8, ammonia at 0.25–0.5 mg/L; nitrites at 0.25 mg/L; and nitrates at 20–40 mg/L.

Tissue samples, including gill, heart, muscle, lymphoid tissue, hepatopancreas, and stomach, were dissected and stored in RNAlater solution (Thermo Fisher Scientific, Schwerte, Germany) at −80 °C until further analysis. Hemocytes were isolated by extracting hemolymph from the ventral region (above the first abdominal segment) using a sterilized syringe preloaded with an anticoagulant Alsever’s solution (Sigma-Aldrich, St. Louis, MO, USA) in a 1:1 ratio. Hemolymph was immediately centrifuged at 8000× *g* for 15 min at 4 °C after collection. Ethical approval was not required for this study because experiments were conducted using an invertebrate species not subject to mandatory institutional animal ethics review under current regulations.

### 2.2. Total RNA Extraction and cDNA Cloning

Total RNA was extracted from the tissues stored in RNAlater using a commercial RNA extraction kit (Bioneer, Daejeon, Republic of Korea) with genomic DNA elimination step according to the manufacturer’s instructions. RNA quality and quantity were assessed using the NP-80 Nanophotometer (Implen, Munich, Germany). Only RNA samples with A260/A280 and A260/A230 ratios greater than 1.9 were used for subsequent procedures.

To identify the complete *Pv*Prx6 cDNA sequence, rapid amplification of cDNA ends (RACE) was carried out with the SMARTer RACE 5′/3′ Kit (Takara Bio Inc., Kusatus, Japan) and the Invitrogen 3′ RACE System (Thermo Fisher Scientific, Waltham, MA, USA) according to the manufacturers’ instructions, using 1 μg of pooled total RNA prepared by combining equal amounts of RNA extracted from each tissue. The primer sets for the primary and nested PCR for the RACE reactions were designed based on partial nucleotide sequences found in our local *P. vannamei* transcriptomic database (Table 1), which showed homology to orthologs in crustacean species, including *Scylla paramamosain* (accession no. JX133231) and *Eriocheir sinensis* (accession no. EU626070), available in the NCBI GenBank database. The primary touch-down PCRs for both 5′ and 3′ RACE were performed with the initial 5 cycles of 95 °C for 30 s, 70 °C for 30 s, and 72 °C for 2 min followed by 5 cycles of 95 °C for 30 s, 68 °C for 30 s, and 72 °C for 2 min; and final 20 cycles of 95 °C for 30 s, 65 °C for 30 s, and 72 °C for 2 min. Nested PCR for both 5′- and 3′-RACE was performed using the primary RACE-PCR products under the following conditions: initial denaturation at 95 °C for 5 min, followed by 30 cycles of 95 °C for 30 s, 60 °C for 30 s, and 72 °C for 1 min, with a final extension at 72 °C for 5 min. The nested RACE products were cloned into the pTOP V2 vector (Enzynomics, Daejeon, Republic of Korea) and verified by sequencing in both directions. To further validate the complete open reading frame (ORF), an additional PCR was performed using a pair of gene-specific primers designed to encompass the start and stop codons (Table 1). The sequencing results were assembled using BioEdit v7.7.1 program to determine the complete nucleotide sequence of *Pv*Prx6 cDNA.

### 2.3. In Silico Sequence Characterization and Molecular Phylogeny

The putative open reading frame (ORF) of *Pv*Prx6 cDNA and its deduced amino acid sequence were predicted using the NCBI ORF finder tool (https://www.ncbi.nlm.nih.gov/orffinder, accessed on 18 May 2024). The Simple Modular Architecture Research Tool (SMART; http://smart.embl-heidelberg.de/, accessed on 18 May 2024) and the InterProScan (InterPro; https://www.ebi.ac.uk/interpro/, accessed on 18 May 2024) were used to analyze the domain architectures and motif sequences of the *Pv*Prx6 protein [36,37]. The theoretical isoelectric point (pI) and molecular weight (MW) of the *Pv*Prx6 protein were computed using the ExPASy pI/Mw tool (https://web.expasy.org/compute_pi/, accessed on 18 May 2024) [38]. The potential subcellular localization of *Pv*Prx6 protein was analyzed using CELLO v.2.5 (http://cello.life.nctu.edu.tw/, accessed on 18 May 2024) [39]. Multiple sequence alignment (MSA) of the *Pv*Prx6 protein with other orthologs was conducted using Clustal Omega (http://www.ebi.ac.uk/Tools/msa/clustalo/, accessed on 18 May 2024).

A molecular phylogenetic tree was constructed using the Maximum Likelihood (ML) method to infer the evolutionary relationship of *Pv*Prx6 with its orthologs using Molecular Evolutionary Genetics Analysis (MEGA) 11 software [40]. The Prx protein sequences used for MSA and phylogenetic analysis were retrieved from the NCBI GenBank database, and their accession numbers are listed in Supplementary Table S1. Representative Prx6 orthologs from vertebrates and invertebrates were selected based on sequence completeness and annotation reliability. The reliability of the phylogenetic tree was evaluated using 1000 bootstrap replications. Typical 2-Cys Prxs (Prx1 from *P. monodon* and *Homo sapiens*), along with *P. vannamei* Prx4 (*Pv*Prx4), were included for comparative purposes to distinguish the 1-Cys Prx clade, rather than as strict evolutionary outgroups.

### 2.4. Three-Dimensional (3D) Structural Modeling

To predict the 3D structure of the *Pv*Prx6 protein, AlphaFold2 was utilized via the Neurosnap platform (https://neurosnap.ai/, accessed on 18 May 2024) [41]. The modeling process incorporated ColabFold for efficient structural assembly [42]. MSA was conducted using the mmseqs2_uniref_env mode to capture deep evolutionary information [43]. The number of recycles was set to 5 to refine the structural stability of the predicted *Pv*Prx6 model. Structural reliability was assessed through Predicted Local Distance Difference Test (pLDDT) and Predicted Aligned Error (PAE) scores. The final structural visualization and mapping of the catalytic triad (active site) were performed using the PyMOL Molecular Graphics System (Version 3.0, Schrödinger, LLC, Portland, OR, USA).

### 2.5. Expression Analysis of PvPrx6 mRNA

To examine the basal expression levels of *Pv*Prx6 mRNAs, seven tissues (gill, heart, hemocytes, hepatopancreas, lymphoid organ, muscle, and stomach) were surgically obtained from nine individuals as described in Section 2.1. Three biological replicates were prepared for each tissue by pooling equal amounts of tissue from three individuals. Each pooled sample was considered as one biological replicate.

To investigate the transcriptional regulation of *Pv*Prx6 mRNA in response to immune challenges, shrimp were randomly assigned to four 40 L tanks (30 individuals per tank), including control and experimental groups. Immune challenges were performed by injecting 10 µL of lipopolysaccharide (LPS) (*Escherichia coli* 0111: B4, Sigma-Aldrich, St. Louis, MO, USA), peptidoglycan (PGN) (*Staphylococcus aureus*, Sigma-Aldrich, St. Louis, MO, USA), and polyinosinic-polycytidylic acid (poly I: C) (Sigma-Aldrich, St. Louis, MO, USA) suspended in sterilized phosphate-buffered saline (PBS, pH 7.4) at a concentration of 2 µg/µL into the abdominal segments III and IV of each shrimp, corresponding to an actual dose of 20 µg per shrimp. The non-stimulated control group received an equivalent volume of PBS. At each sampling time point (0, 6, 12, 24, and 48 h post-injection), three shrimp were randomly selected and pooled to generate one biological replicate, and the immune challenge experiment was conducted in three independent trials, resulting in three pooled biological replicates per treatment group (*n* = 3).

Total RNA was extracted as described in Section 2.2, and cDNA was subsequently synthesized from 1 µg total RNA using AccuPower RT PreMix with oligo(dT) primer (Bioneer, Daejeon, Republic of Korea), according to the manufacturer’s instructions. The synthesized cDNA was diluted 10-fold prior to use as a template for real-time quantitative PCR (RT-qPCR) analysis. RT-qPCR was performed using the LightCycler 480 Real-Time PCR system (Roche Applied Science, Penzberg, Germany). Each reaction was carried out in a total volume of 20 μL, containing 10 μL TOPreal qPCR 2X PreMix (Enzynomics, Daejeon, Republic of Korea), 2 μL of diluted cDNA, 0.4 μL of each primer (10 pmol/μL) and 7.2 μL nuclease-free water, and amplification was performed under the following cycling conditions: 45 cycles of denaturation at 95 °C for 10 s, annealing at 60 °C for 15 s, and extension at 72 °C for 15 s. A melt curve analysis was conducted over a temperature range of 65–97 °C to verify amplification specificity, and single amplicon formation was confirmed by the presence of a single peak. RT-qPCR analyses were performed in technical triplicate for each biological replicate. The *P. vannamei* elongation factor 1α (*Pv*EF1α) gene was used as the internal control for normalization [44], and primer sequences are listed in Table 1. *Pv*EF1α was selected as a reference gene based on its widespread use in RT-qPCR studies of *P. vannamei*, particularly in immune challenge and pathogen-related experiments [45,46,47]. Amplification efficiencies were estimated to be 1.91 for *Pv*Prx6 and 1.98 for *Pv*EF1α. Relative gene expression levels were calculated using the 2^−ΔΔCt^ method [48], and results are presented as the mean ± standard deviation (SD). Statistical analysis was performed using one-way analysis of variance (ANOVA) followed by Tukey’s honestly significant difference (HSD) multiple comparison test using SPSS v25 software. Differences were considered statistically significant at *p* < 0.05.

### 2.6. Production of Recombinant Maltose-Binding Protein-Fused PvPrx6 Protein (MBP-rPvPrx6) in E. coli

The complete ORF sequence encoding the full-length *Pv*Prx6 protein was amplified from the cDNA using gene-specific primers with *BamHI* and *HindIII* restriction sites (Table 1). The PCR product was cloned into the pMAL-c5X vector, which has a maltose-binding protein (MBP) tag for enhanced solubility and purification. The recombinant plasmid (pMAL-c5X-*Pv*Prx6) was transformed into *E. coli* DH5α competent cells and positive transformants were picked and sequenced. The verified recombinant plasmid was then transformed into *E. coli* Rosetta (DE3) cells (Qiagen, Hilden, Germany) and cultured in 1 L of LB medium (1% tryptone, 0.5% yeast extract, and 1% NaCl) containing 50 μg/mL ampicillin at 37 °C with shaking at 200 rpm until the OD_600_ reached 0.6. Protein expression was induced by adding 0.4 mM isopropyl β-d-1-thiogalactopyranoside (IPTG), followed by incubation at 20 °C for 18 h. The induced cells were harvested by centrifugation at 4 °C (4000 rpm for 30 min), washed three times with 10 mL of ice-cold PBS (pH 7.4), and resuspended in 5 mL of ice-cold PBS. Cells were lysed by sonication on ice (amplitude 30%, 30s on/5 min off cycles for 5 cycles), and the lysate was centrifuged at 20,000× *g* for 20 min at 4 °C to separate soluble and insoluble fractions. The recombinant MBP-r*Pv*Prx6 protein was purified following the manufacturer’s protocol (New England Biolabs, Beverly, MA, USA). The soluble fraction was diluted fivefold in column buffer (20 mM Tris-HCl, 200 mM NaCl, pH 7.4) and applied to an amylose resin column pre-equilibrated with column buffer, followed by washing with the same buffer to remove non-specifically bound proteins. The recombinant protein was then eluted using column buffer supplemented with 10 mM maltose. Successful expression and purification of MBP-r*Pv*Prx6 were confirmed by 12% SDS-PAGE, and the concentration was determined by a Bradford assay (Bio-Rad Laboratories, Seoul, Republic of Korea).

### 2.7. Metal-Catalyzed Oxidation (MCO) Assay

The MCO assay was used to investigate the antioxidant activity of r*Pv*Prx6 against ROS-induced DNA damage, following a method previously described with slight modifications [49,50]. Briefly, a 20 μL reaction mixture containing 3.3 mM dithiothreitol (DTT), 16.5 μM FeCl_3_, and varying concentrations of purified MBP-r*Pv*Prx6 (37.5–300 μg/mL) was incubated at 37 °C for 3 h. Then, 1 μg of pUC19 supercoiled DNA was added to each reaction mixture and incubated at 37 °C for another 3 h. Finally, the reaction mixtures were analyzed by electrophoresis on a 1% agarose gel. The reactions containing MBP (300 μg/mL) or BSA (300 μg/mL) instead of purified MBP-r*Pv*Prx6 were used as controls to demonstrate the absence of antioxidant activity. The MCO-based DNA protection assay was used as an initial in vitro approach to evaluate whether r*Pv*Prx6 could exert antioxidant-like protective effects under oxidative conditions, as previously applied in characterization studies of Prx-related proteins [49,50,51].

## 3. Results

### 3.1. Molecular Identification and Sequence Characterization of PvPrx6

**Figure 1:**
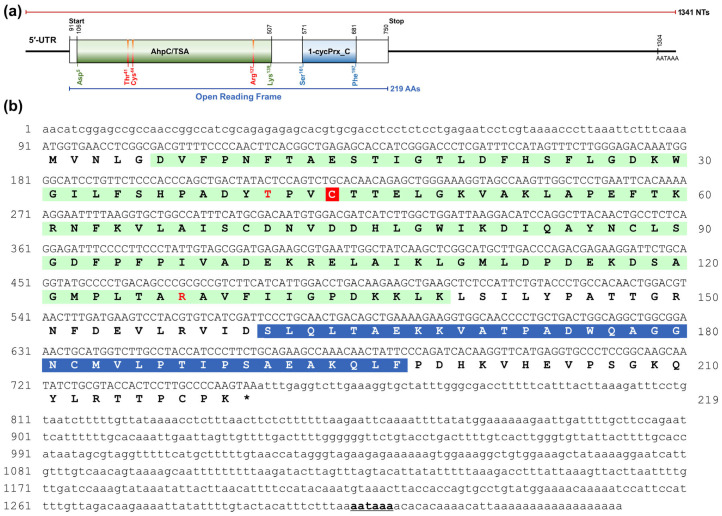
Structure and sequence analysis of *Pv*Prx6. (**a**) Schematic representation of the *Pv*Prx6 cDNA and its domain architecture. The positions of the UTRs, ORF, and conserved domains (AhpC/TSA and 1-cysPrx_C) are indicated with the catalytic triad (Thr^41^, Cys^44^, Arg^127^). (**b**) Nucleotide and deduced amino acid sequences of *Pv*Prx6. The AhpC/TSA and 1-Cys Prx domains are shaded in light green and blue, respectively. Red letters indicate the residues of the catalytic triad, and the peroxidatic cysteine (*C_P_*) is highlighted with a red background. The asterisk (*) indicates a translation stop. The black bold and underlined letters (aataaa) indicate the typical polyadenylation signal.

A cDNA clone showing high sequence homology to other crustacean Prx6 was initially identified from our local *P. vannamei* transcriptome database. Based on the initial sequence, the full-length cDNA of *Pv*Prx6 was successfully identified using RACE. The complete *Pv*Prx6 cDNA was 1341 bp in length, comprising a 90 bp 5′-untranslated region (5′-UTR), an ORF of 660 bp (including the TAA stop codon), and a 591 bp 3′-UTR (Figure 1a). A typical polyadenylation consensus signal was identified in the 3′-UTR. The ORF encoded a protein of 219 amino acids (AAs) (Figure 1b).

The theoretical MW and pI of the deduced *Pv*Prx6 protein were 24.1 kDa and 5.73, respectively. Subcellular localization analysis using the CELLO v.2.5 server predicted that *Pv*Prx6 is predominantly located in the cytoplasm with a reliability score of 2.746, indicating a predicted cytoplasmic localization.

Domain architecture analysis using InterProScan and SMART revealed that *Pv*Prx6 contained a 1-Cys PRX domain (Gly^4^–Pro^216^) and was classified into the 1-Cys PRX subfamily. The protein possessed two conserved structural signatures, including the alkyl hydroperoxide reductase subunit C/thiol-specific antioxidant (AhpC/TSA) domain and the C-terminal 1-Cys Prx (1-cysPrx_C) domain. Within the AhpC/TSA domain, a peroxidase catalytic triad consisting of Thr^41^, Cys^44^, and Arg^127^ was identified, with Cys^44^ representing the conserved peroxidatic cysteine (*C_P_*) (Figure 1b).

### 3.2. Multiple Sequence Alignment (MSA) and Phylogenetic Analysis

**Figure 2:**
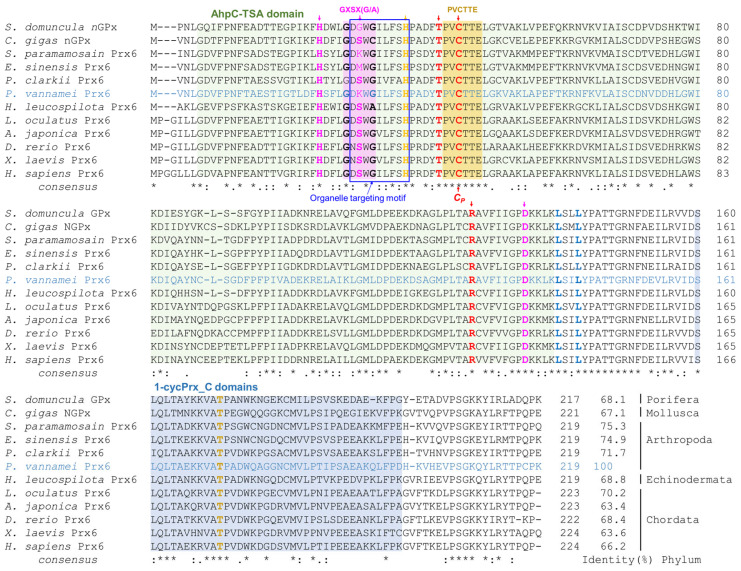
MSA of *Pv*Prx6 with various Prx6 orthologs. The AhpC/TSA domain and 1-Cys Prx domain are indicated with light green and light blue shaded backgrounds, respectively. The PVCTTE motif is highlighted with an orange shaded background. Red bold letters marked with downward arrows represent the core peroxidase catalytic triad (Thr^41^, Cys^44^, and Arg^127^), while with the orange bold letter with downward arrow identifies the His^36^ residue essential for peroxidatic cysteine (*C_P_*) activation. The brown bold letter indicates the Thr^172^ phosphorylation site. The blue box outlines the N-terminal motif region (Asp^28^–Pro^36^), which encompasses the GXSX(G/A) lipase motif. Pink bold letters with downward arrows represent the PLA_2_-associated residues, including His^23^ and the residues within the lipase motif. The blue bold letters (Leu^140^ and Leu^143^) indicate conserved hydrophobic residues likely contributing to structural stability. Asterisks (*), colons (:), and periods (.) indicate fully conserved residues, strongly similar residues, and weakly similar residues, respectively.

MSA of the *Pv*Prx6 with Prx6 orthologs from representative species was performed (Figure 2). All sequences shared the characteristic domain architecture of Prx6 proteins, including the AhpC/TSA and 1-Cys Prx domains. The conserved ^42^PVCTTE^47^ motif, a defining feature of the Prx6 subfamily, was strictly conserved across all species. Detailed residue-level information is presented in Figure 2.

In the N-terminal region, an organelle-associated motif encompassing the GXSXG/A lipase motif was identified. The peroxidase catalytic triad (Thr^41^, Cys^44^, and Arg^127^) and residues corresponding to the reported PLA_2_-associated motif were generally conserved across species, with minor lineage-specific variations. Sequence identity analysis based on Supplementary Table S1 showed that *Pv*Prx6 shares the highest identity with crustacean orthologs, including *S. paramamosain* and *E. sinensis*.

**Figure 3:**
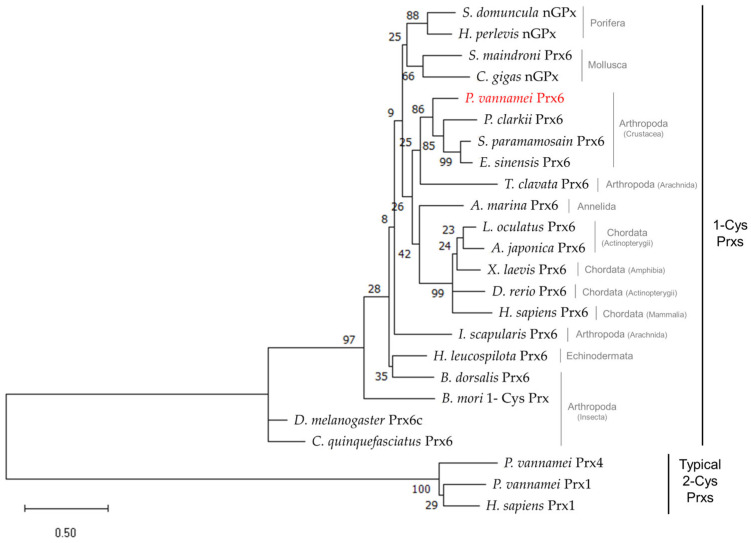
ML phylogenetic tree of *Pv*Prx6 and representative Prx orthologs. The tree was reconstructed based on 21 representative Prx sequences with 1000 bootstrap replicates. Numbers at each node represent the bootstrap support values (%), and the scale bar indicates the evolutionary distance (0.5 substitutions per site). *Pv*Prx6 is nested within the 1-Cys Prx (Prx6 subfamily) clade, forming a distinct cluster with other decapod species. 2-Cys Prxs (Prx1 and Prx4) were included for comparative purposes to distinguish the 1-Cys Prx clade.

To investigate the evolutionary relationship, an ML phylogenetic tree was constructed (Figure 3). The results showed that *Pv*Prx6 was grouped within the monophyletic 1-Cys Prx clade and clearly separated from the typical 2-Cys Prx group (Prx1 and Prx4) with a high bootstrap support. Within this clade, decapod species formed a well-supported cluster. *Pv*Prx6 clustered with the freshwater crayfish, *P. clarkii*, followed by the marine crab species.

### 3.3. 3D Structural Modeling and Comparative Analysis of PvPrx6

**Figure 4:**
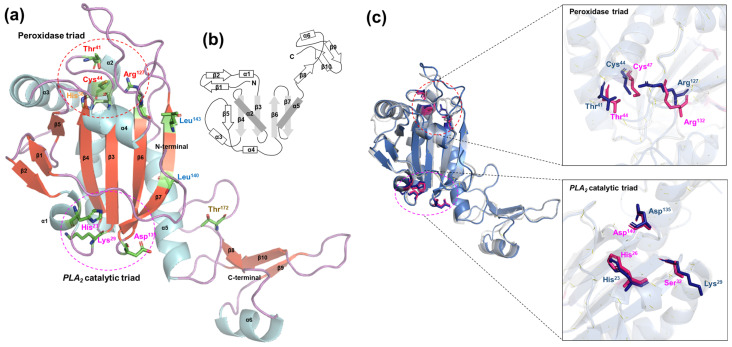
3D structural modeling of *Pv*Prx6. (**a**) Predicted 3D structure of *Pv*Prx6 that was generated via AlphaFold2, with functional residues and conserved motifs highlighted based on MSA results. (**b**) Topology diagram of *Pv*Prx6. The arrangement of seven β-strands flanked by six α-helices reveals a canonical thioredoxin-like fold. (**c**) Structural superimposition with human Prx6 (PDB ID: 1PRX). Left: Global alignment showing high structural homology (RMSD = 0.845 Å). Right-up: Close-up superimposition of the peroxidase triad of *Pv*Prx6 (Thr^41^, Cys^44^, and Arg^127^) with that of human Prx6 (Thr^44^, Cys^47^, and Arg^132^). Right-down: Close-up of the PLA_2_-associated residues (His^23^, Lys^29^, and Asp^135^), showing a comparable spatial arrangement despite the Ser-to-Lys substitution.

The tertiary structure of *Pv*Prx6 was predicted using AlphaFold2 to examine the spatial arrangement of predicted active-site-related regions (Figure 4a). The predicted model exhibited high reliability, with an average pLDDT score of 96.89, indicating a high-confidence structural prediction. As shown in the topology diagram (Figure 4b), *Pv*Prx6 adopts a thioredoxin-like fold consisting of central β-strands flanked by α-helices [52].

The peroxidase catalytic residues were located in positions similar to those observed in human Prx6, forming a conserved catalytic region. These residues were positioned in close proximity within the predicted active site. The residues corresponding to the reported PLA_2_-associated motif also showed a similar spatial arrangement despite sequence variation. This structural similarity does not imply functional equivalence and requires experimental validation. Conserved residues such as Leu^140^ and Leu^143^ were located within the hydrophobic core of the protein, indicating a structural role in maintaining protein stability. Overall, the predicted structural features are consistent with those reported for other Prx6 proteins.

### 3.4. Tissue Distribution and Transcriptional Response of PvPrx6 to Immune Challenges

**Figure 5:**
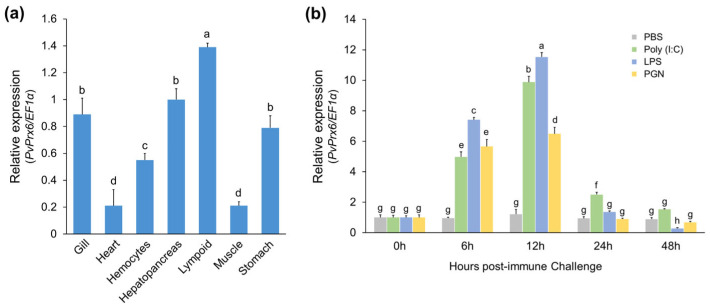
Tissue distribution and transcriptional profiles of *Pv*Prx6. (**a**) Basal mRNA levels of *Pv*Prx6 across various tissues in healthy *Penaeus vannamei*. (**b**) Temporal mRNA expression in the lymphoid organ following challenges with PBS, Poly(I:C), LPS, and PGN. Data are expressed as mean ± SD (*n* = 3). Different letters indicate significant differences among all experimental groups (*p* < 0.05) based on one-way ANOVA followed by Tukey’s HSD post hoc test.

To examine the expression patterns of *Pv*Prx6, its basal expression across various tissues and its transcriptional responses under immune stimulation were investigated using RT-qPCR. The tissue distribution analysis revealed that *Pv*Prx6 transcripts were ubiquitously detected in all examined tissues of *P. vannamei*, although their expression levels varied significantly (Figure 5a). The highest basal expression was observed in the lymphoid organ, followed by the hepatopancreas and gills. Moderate expression levels were noted in the stomach and hemocytes, while the heart and muscle exhibited relatively low expression.

The transcriptional response of *Pv*Prx6 in the lymphoid organ was further analyzed following challenges with various PAMPs, including Poly(I:C), LPS, and PGN (Figure 5b). In the early phase of the immune response (6 h post-injection), *Pv*Prx6 transcript levels were significantly upregulated in all PAMP-challenged groups. Notably, the LPS-challenged group exhibited a more robust induction (7.41-fold) compared to the Poly(I:C) (4.97-fold) and PGN (5.66-fold) groups. At 12 h post-injection, all challenged groups reached their peak expression levels, with LPS inducing the strongest response, followed by Poly(I:C) and PGN (*p* < 0.05). As the immune response progressed to the late phase (48 h), expression patterns diverged depending on the stimulant. *Pv*Prx6 mRNA levels in the bacterial mimic-challenged groups (LPS and PGN) gradually declined toward the baseline. *Pv*Prx6 expression decreased below control levels at 48 h post-LPS injection. Conversely, the Poly(I:C)-challenged group maintained an elevated expression level of 1.53-fold.

### 3.5. Recombinant Production of PvPrx6 and Its Antioxidant Activity

**Figure 6:**
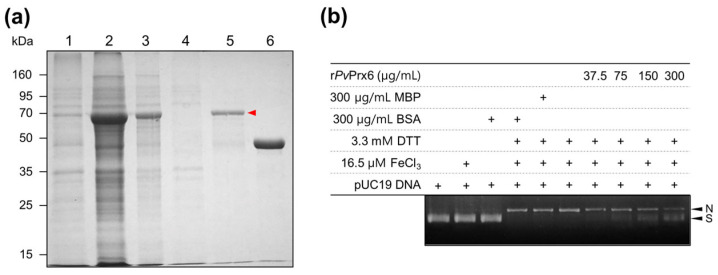
Recombinant production and DNA protection activity of r*Pv*Prx6. (**a**) SDS-PAGE analysis. Lane 1: non-induced *E. coli* lysate; Lane 2: IPTG-induced whole cell lysate; Lane 3: soluble fraction; Lane 4: insoluble fraction; Lane 5: purified MBP-r*Pv*Prx6 (red arrowhead); Lane 6: purified MBP tag. (**b**) Protective effect of r*Pv*Prx6 against ROS-induced DNA damage. pUC19 supercoiled DNA was incubated with FeCl_3_ and DTT in the presence of r*Pv*Prx6 (37.5–300 µg/mL). MBP (300 µg/mL) or BSA (300 µg/mL) served as negative controls. Arrowheads indicate supercoiled (S) and nicked (N) DNA forms.

For biochemical characterization, *Pv*Prx6 was expressed as an MBP-fused protein in *E. coli*. SDS-PAGE analysis confirmed the successful induction and purification of MBP-r*Pv*Prx6, yielding a distinct band at approximately 70 kDa (Figure 6a). This size aligns with the combined predicted molecular weight of r*Pv*Prx6 (26.1 kDa) and the MBP tag (42.5 kDa). The recombinant protein was predominantly localized in the soluble fraction, allowing purification for subsequent assays.

The antioxidant activity of r*Pv*Prx6 was assessed by its ability to protect pUC19 supercoiled plasmid DNA from oxidative damage induced by the metal-catalyzed oxidation (MCO) system (Figure 6b). In the complete MCO system (Fe^3+^/DTT), supercoiled DNA was converted to nicked forms, indicating increased DNA damage. The addition of r*Pv*Prx6 (37.5–300 µg/mL) inhibited DNA cleavage in a clear dose-dependent manner. In contrast, negative controls containing MBP or BSA (300 µg/mL) did not show any protective effect. These results indicate that r*Pv*Prx6 can protect plasmid DNA from oxidative damage under the experimental conditions.

## 4. Discussion

Aquatic organisms are frequently exposed to oxidative stress caused by ROS, which arise from environmental fluctuations and physiological processes [2]. In this context, structural characterization of antioxidant enzymes provides important insight into redox regulation in marine invertebrates. In this study, *Pv*Prx6 from the whiteleg shrimp *P. vannamei* was identified as a member of the 1-Cys Prx6 subfamily, which has been reported in other species to be associated with peroxidase activity and PLA_2_-related function [16,18,20,53].

Sequence comparison and 3D structural analysis confirmed that *Pv*Prx6 contains conserved domains, including the AhpC/TSA and the 1-Cys Prx domain, which are commonly observed in Prx proteins [15,54]. The predicted 3D structure adopts a typical thioredoxin-like fold observed in Prx6 proteins, suggesting conservation of the typical Prx6 structural fold rather than confirming functional equivalence [14,55]. The presence of the catalytic triad (Thr^41^, Cys^44^, and Arg^127^) and the conserved His^36^ residue indicates structural similarity to other Prx6 proteins [54,56]. The conservation of these residues suggests that *Pv*Prx6 may retain catalytic features typical of Prx6 proteins, although direct enzymatic activity was not examined in this study.

In contrast, a variation was observed in the PLA_2_-related motif. *Pv*Prx6 contains a putative catalytic center (His^23^, Lys^29^, and Asp^135^), in which the nucleophilic Ser residue is replaced by Lys [20,57]. Structural superimposition analysis showed that Lys^29^ occupies a spatial position comparable to Ser^32^ in human Prx6 [54,56]. This structural similarity does not imply functional equivalence and requires experimental validation. Thus, Lys^29^ may represent a residue-level divergence occurring within an otherwise conserved structural framework.

Phylogenetic analysis further supports this interpretation. *Pv*Prx6 clusters within a monophyletic clade of decapod orthologs, showing close association with *P. clarkii*. Additional motif variations observed among crustacean species, including *S. paramamosain* and *E. sinensis*, suggest diversification within a conserved Prx6 lineage. This pattern may reflect sequence variation within a conserved structural framework, although its functional significance remains unresolved.

The physiological relevance of *Pv*Prx6 was examined through tissue distribution and immune challenge experiments. *Pv*Prx6 transcripts were detected in all examined tissues, with relatively high expression levels in the lymphoid organ and hepatopancreas. In decapods, these tissues are associated with immune function and metabolic regulation. The elevated basal expression of *Pv*Prx6 in these tissues suggests a potential role in antioxidant processes associated with immune activity and metabolism, although direct functional evidence was not obtained in this study.

Following immune challenge, *Pv*Prx6 expression was significantly upregulated in response to all tested PAMPs, including LPS, poly(I:C), and PGN, with peak expression observed at 12 h post-injection. This induction pattern may be associated with oxidative responses occurring during immune activation. Previous studies have shown that antioxidant-related genes in shrimp can be modulated during immune activation [50,58,59]. Although the regulatory pathways controlling *Pv*Prx6 were not examined in this study, the observed expression pattern is consistent with a possible role in immune-associated oxidative responses.

The antioxidant activity of recombinant *Pv*Prx6 (r*Pv*Prx6) was evaluated using a DNA protection assay. r*Pv*Prx6 inhibited hydroxyl radical-induced DNA damage in a dose-dependent manner. These findings indicate that r*Pv*Prx6 can protect DNA from oxidative damage under the experimental conditions, but do not directly demonstrate enzymatic peroxidase activity or quantitative ROS scavenging capacity. Hydroxyl radicals are highly reactive ROS that can damage nucleic acids, and similar DNA-protective effects have been reported in other Prx6 orthologs [49,50,51].

The DNA protection assay provides a qualitative assessment of antioxidant activity. Further biochemical analyses, including H_2_O_2_ reduction assays, lipid peroxidation assays, and direct peroxidase activity measurements, would be required to fully characterize the enzymatic function of *Pv*Prx6. In addition, *Pv*EF1α is widely used as a reference gene in shrimp RT-qPCR studies; however, the use of a single reference gene may introduce bias under certain experimental conditions. Future studies should include validation of multiple candidate reference genes to ensure accurate normalization.

Taken together, the results suggest that *Pv*Prx6 retains the conserved structural characteristics of Prx6 proteins and may contribute to antioxidant defense in *P. vannamei*. However, its precise biochemical functions and physiological roles in redox regulation and immune-associated oxidative stress responses require further validation through quantitative assays and in vivo studies.

## 5. Conclusions

This study identified and characterized a 1-Cys peroxiredoxin 6 gene (*Pv*Prx6) from *P. vannamei*. *Pv*Prx6 encodes a 219-amino-acid protein containing conserved AhpC/TSA and 1-Cys Prx domains and adopts a canonical thioredoxin-like fold. Structural modeling suggested the presence of a conserved peroxidase catalytic triad (Thr^41^, Cys^44^, and Arg^127^) and indicated that Lys^29^ occupies a spatial position corresponding to Ser^32^ in mammalian Prx6. However, this structural similarity does not imply functional equivalence and requires experimental validation. Phylogenetic analysis further indicated diversification at the PLA_2_-associated motif within the decapod lineage while preserving the overall structural framework. *Pv*Prx6 was ubiquitously expressed, with the highest transcript levels observed in the lymphoid organ and hepatopancreas, and was significantly induced following LPS, poly(I:C), and PGN challenges. Recombinant *Pv*Prx6 exhibited dose-dependent protection against hydroxyl radical-mediated DNA damage in vitro. These results indicate that r*Pv*Prx6 can protect DNA from oxidative damage under the experimental conditions. Collectively, these findings suggest that *Pv*Prx6 shares conserved structural characteristics with other Prx6 proteins and may be associated with antioxidant defense in *P. vannamei*. However, further studies are required to experimentally validate its enzymatic activity and in vivo functional roles.

## 6. Patents

The authors declare that no patents have been filed or granted based on the findings of this study.

## Figures and Tables

**Table 1:** Primer sequences used in this study.

Primer Name	Sequences (5′-3′)	Purpose
5-GSP	AGACCATGCAGTTTCCGCCAGCCT	5′ RACE
5-GSP_nested	ACGACCAGTTGTGGCAGGGTACAGA	
*Takara_Longup* ^a^	*CTAATACGACTCACTATAGGGCAAGCAGTGGTATCAACGCAGAGT*	
*Takara_Shortup* ^a^	*CTAATACGACTCACTATAGGGC*	
3-GSP	AGCACCATCGGGACCCTCGATTTCC	3′ RACE
3-GSP-nested	AAGCTCGGCATGCTTGACCCAGACG	
*AUAP* ^a^	*GGCCACGCGTCGACTAGTAC*	
*Pv*Prx6-Exp-F ^b^	gagaga**GGATCC**ATGGTGAACCTCGGCGAC	Full-length ORF cloning and expression vector construction
*Pv*Prx6-Exp-R ^b^	gagaga**AAGCTT**TTACTTGGGGCAAGGAGT	
*Pv*Prx6-qF	ATGGTCTTGCCTACCATCCCTTCT	RT-qPCR analysis
*Pv*Prx6-qR	AAGGTCGCCCAAATAGCACCTTTC	
*Pv*EF1α-qF	TCGCCGAACTGCTGACCAAGA	
*Pv*EF1α-qR	CCGGCTTCCAGTTCCTTACC	

^a^ Italics indicate the universal primers provided by the commercial SMARTer RACE 5′/3′ Kit (Takara Bio) and Invitrogen 3′ RACE System. ^b^ Lowercase letters indicate the protective bases, while bold and underlined letters represent the restriction enzyme recognition sites for *BamHI* and *HindIII*. These sites were incorporated for in-frame ligation into the pMAL-c5X expression vector.

## Data Availability

The nucleotide sequence of *Pv*Prx6 has been deposited in the NCBI GenBank database under accession number PP680572. All other data generated or analyzed during this study are included in this article and its Supplementary Materials. Additional raw data are available from the corresponding author upon reasonable request.

## Supplementary Materials

The following supporting information can be downloaded at: https://www.mdpi.com/article/10.3390/genes17040428/s1, Table S1: Species and GenBank accession number of Prx sequences used for multiple sequence alignment and phylogenetic analysis.

## Abbreviations

The following abbreviations are used in this manuscript:

AHPND	Acute Hepatopancreatic Necrosis Disease
AhpC/TSA	Alkyl hydroperoxide reductase C/Thiol-specific antioxidant
IMD	Immune deficiency
JAK/STAT	Janus kinase/Signal transducer and activator of transcription
LPS	Lipopolysaccharide
MCO	Metal-catalyzed oxidation
MSA	Multiple sequence alignment
PAMP	Pathogen-associated molecular pattern
PGN	Peptidoglycan
PLA_2_	Phospholipase A_2_
Prx	Peroxiredoxin
Prx6	Peroxiredoxin 6
*Pv*Prx6	*Penaeus vannamei* Peroxiredoxin 6
RACE	Rapid amplification of cDNA ends
RMSD	Root mean square deviation
ROS	Reactive oxygen species
RT-qPCR	Reverse transcription quantitative polymerase chain reaction
WSSV	White spot syndrome virus
