# Supplementary material for: Molecular Characterization and In Vitro Functional Analysis of a 1-Cys Peroxiredoxin 6 from the Whiteleg Shrimp Penaeus vannamei
Source: Genes (Basel). 2026 Apr 6;17(4):428. doi: 10.3390/genes17040428 (PMC13116241; doi:10.3390/genes17040428)
Supplement: Supplementary file 1 [file genes-17-00428-s001.zip › genes-4193284-supplementary.pdf]

Supplementary Table S1. Species and GenBank accession number of Prx sequences used for multiple sequence alignment and phylogenetic analysis.

Phyla	Species	Type	Accession No.	Length (AAs)	Identity (%)	Similarity (%)
Porifera	*Suberites domuncula*	nGPx[a]	CAC38779.1	217	68.1	82.3
	Hymeniacidon perlevis	nGPx	ABB91779.1	218	68.2	83.3
Annelida	*Arenicola marina*	Prx6	AAY96294.1	220	67.6	80.8
Mollusca	*Sepiella maindroni*	Prx6	AEI52300.1	219	66.5	79.2
	Crassostrea gigas	nGPx	CAK22382.1	221	67.1	78.4
Arthropoda (Crustacea)	*Scylla paramamosain*	Prx6	AFP89581.1	219	75.3	89.6
	Eriocheir sinensis	Prx6	ACF35639.1	219	74.9	88.7
	Procambarus clarkii	Prx6	XP_045618654.1	219	71.7	88.7
	Penaeus monodon	Prx1	AQW41372.1	198	29.6	45.2
	Penaeus vannamei	Prx4	AET36895.1	245	25.8	38.2
		Prx6	This study	219	100	100
Arthropoda (Arachnida)	*Trichonephila clavata*	Prx6	GFR31471.1	220	66.5	76.4
	Ixodes scapularis	Prx6	KAM7305231.1	221	65.0	76.2
Arthropoda (Insecta)	*Drosophila melanogaster*	Prx6c	AAF58799.1	220	52.1	68.2
	Bactrocera dorsalis	Prx	JAC48611.1	224	63.6	73.1
	Bombyx mori	1-Cys Prx	ABV32570.1	223	63.5	77.4
	Culex quinquefasciatus	Prx6	XP_001861525.2	221	50.0	70.2
Echinodermata	*Holothuria leucospilota*	Prx6	KAJ8039277.1	219	68.8	81.9
Chordata (Actinopterygii)	*Lepisosteus oculatus*	Prx6	XP_015211473.2	223	70.2	79.7
	Anguilla japonica	Prx6	ALG02507.1	223	63.4	80.2
	Danio rerio	Prx6	AAH59671.1	222	68.4	76.8
Chordata (Amphibia)	*Xenopus laevis*	Prx6	AEM44543.1	224	63.6	77.2
Chordata (Mammalia)	*Homo sapiens*	Prx6	KAI4083970.1	224	66.2	78.2
	Homo sapiens	Prx1	KAI4080428.1	199	30.2	47.4

[a]The Prx6 from *S. domuncula*, *H. perlevis*, and *C. gigas* were designated as nGPX (non-selenium glutathione peroxidase).
